# Australian Headache Epidemiology Data (AHEAD): a pilot study to assess sampling and engagement methodology for a nationwide population-based survey

**DOI:** 10.1186/s10194-024-01773-8

**Published:** 2024-05-06

**Authors:** Emma Foster, Zhibin Chen, Claire E Wakefield, Zanfina Ademi, Elspeth Hutton, Timothy J Steiner, Alessandro S Zagami

**Affiliations:** 1 Department of Neuroscience, Central Clinical School, Monash University, 99 Commercial Road, Melbourne, VIC 3004 Australia; 2 Department of Neurology, Alfred Health, Commercial Road, Melbourne, VIC 3000 Australia; 3 Department of Medicine, University of Melbourne, Parkville, VIC 3050 Australia; 4 School of Public Health and Preventive Medicine, Monash University, Melbourne, VIC 3000 Australia; 5 School of Clinical Medicine, Discipline of Paediatrics, UNSW Sydney, Randwick, NSW 2031 Australia; 6 Health Economics and Policy Evaluation Research (HEPER) Group, Centre for Medicine Use and Safety, Faulty of Pharmacy and Pharmaceutical Sciences, Monash University, Parkville, VIC 3052 Australia; 7 Department of Neuromedicine and Movement Science, Norwegian University of Science and Technology, Trondheim, Norway; 8 Division of Brain Sciences, Imperial College London, London, UK; 9 Institute of Neurological Sciences, Prince of Wales Hospital, Randwick, NSW 2031 Australia; 10 School of Clinical Medicine, Faculty of Medicine, University of New South Wales, Sydney, Australia

**Keywords:** Disease burden, Epidemiology, Methodology, HARDSHIP, Headache, Medication overuse headache, Migraine, Patient reported outcomes, Quality of life, Treatment gap, Work productivity

## Abstract

**Background:**

There are no robust population-based Australian data on prevalence and attributed burden of migraine and medication-overuse headache (MOH) data. In this pilot cross-sectional study, we aimed to capture the participation rate, preferred response method, and acceptability of self-report questionnaires to inform the conduct of a future nationwide migraine/MOH epidemiological study.

**Methods:**

We developed a self-report questionnaire, available in hard-copy and online, including modules from the Headache-Attributed Restriction, Disability, Social Handicap and Impaired Participation (HARDSHIP) questionnaire, the Eq. 5D (quality of life), and enquiry into treatment gaps. Study invitations were mailed to 20,000 randomly selected households across Australia’s two most populous states. The household member who most recently had a birthday and was aged ≥ 18 years was invited to participate, and could do so by returning a hard-copy questionnaire via reply-paid mail, or by entering responses directly into an online platform.

**Results:**

The participation rate was 5.0% (*N* = 1,000). Participants’ median age was 60 years (IQR 44–71 years), and 64.7% (*n* = 647) were female. Significantly more responses were received from areas with relatively older populations and middle-level socioeconomic status. Hard copy was the more commonly chosen response method (*n* = 736). Females and younger respondents were significantly more likely to respond online than via hard-copy.

**Conclusions:**

This pilot study indicates that alternative methodology is needed to achieve satisfactory engagement in a future nationwide migraine/MOH epidemiological study, for example through inclusion of migraine screening questions in well-resourced, interview-based national health surveys that are conducted regularly by government agencies. Meanwhile, additional future research directions include defining and addressing treatment gaps to improve migraine awareness, and minimise under-diagnosis and under-treatment.

**Supplementary Information:**

The online version contains supplementary material available at 10.1186/s10194-024-01773-8.

## Background

Worldwide, epidemiological studies have consistently identified migraine and medication-overuse headache (MOH) as highly prevalent and disabling headache disorders [1, 2], but there are no robust population-based Australian data. Australian epidemiological studies of migraine have so far been limited to certain age groups, geographical areas, or care settings [3–5]. Other Australian studies that report migraine prevalence have not been designed with the primary intention of collecting these data, and therefore have limited generalisability across the general population [6–8]. No formal study of MOH has been undertaken in Australia. Extrapolating data from international studies does not accurately reflect Australia’s unique population and healthcare system.

Population-based studies within Australia are therefore needed to determine the scope and scale of headache disorders, especially migraine and MOH, as these are the most likely major public health issues [9]. Such studies are ideally conducted through face-to-face engagement with participants by trained interviewers [10], but this is resource intensive. Our purpose here, prior to undertaking a definitive study, was to assess the feasibility of using mailed questionnaires as a much less costly alternative to establish the prevalence and burden of migraine and probable MOH (pMOH) in Australia.

## Methods

The study design was a cross-sectional enquiry by a questionnaire mailed to addresses selected randomly to be representative of Australia’s population aged 18 years or older. The methodology was informed by the principles and recommendations [10] set out by the Global Campaign against Headache [11]. 

### Aims

The overall purpose was to inform the design, logistic planning, and effective conduct of a future nationwide headache epidemiological study. The aims of this pilot study were to: (1) establish the participation rate, preferred response method (paper-based vs. online), and acceptability of mailed self-report study questionnaires; and (2) provide estimates of prevalence, burden, and treatment gaps to inform power calculations in a future study.

### Selection of postal addresses to represent the general population

We conducted the study in New South Wales and Victoria, the two most populous states in Australia.

We used a two-stage cluster sampling approach to implement equal probability sampling for households. We first sampled 100 local government areas (LGAs) from the 207 LGAs in Victoria and New South Wales using the probability proportional to size with probability minimum replacement sampling method, where the probability of selecting an LGA was proportionate to the total number of private dwellings in the LGA. Depending on the number of private dwellings in the LGA, it was possible that large LGAs would be sampled more than once and each sample was considered an independent cluster. The maximum number of times an LGA could be sampled was restricted by the probability minimum replacement method. In the second stage, 200 individual households in each sampled LGA were selected using simple random sampling method.

HopeWiser, a company providing an Australia Post-accredited address matching approval system, provided the addresses of 20,000 randomly selected households from the study LGAs across Victoria and New South Wales. HopeWiser extracted all valid addresses (i.e., those that had been identified on more than one source for the GeoCoded National Address File dataset), then used software to match these addresses against Australia Post’s Postal Address File (a highly sampled, mature dataset, having been commercially available for > 23 years with monthly updates). The resultant matches were enhanced by markers for ‘residential, ‘non-residential’, ‘deliverable’, and ‘non-deliverable’ provided by Australia Post.

This process captured the vast majority of deliverable residential addresses in Australia with high reliability in terms of completeness and accuracy (< 2% of households use postal services such as roadside mailbags or post office boxes rather than a letter box at their residential address).

### Questionnaire

We used validated instruments where possible for each section of our study questionnaire (Appendix 1).

The core of the present questionnaire utilised modules from the Headache-Attributed Restriction, Disability, Social Handicap and Impaired Participation (HARDSHIP) questionnaire [11], which has already been used in > 20 countries to measure headache prevalence and attributed burden in non-clinical settings [12]. Although only initially validated for face-to-face administration by trained lay interviewers, HARDSHIP has also been adapted as a self-report instrument, in the EUROLIGHT questionnaire [13]. Enquiry into basic demographic data (age, gender, postal/zip code, preferred language, Aboriginal and/or Torres Strait Islander status) was followed by headache screening questions (ever, and in the preceding year) and diagnostic questions based on ICHD-3. We used the Headache-Attributed Lost Time questionnaire (HALT) for capturing headache-attributed lost productivity [14], and the generic health-related quality of life (HR-QOL) EQ-5D-5 L instrument. Further questions addressed healthcare utilisation (headache-related outpatient visits, tests, and emergency department and hospital attendances within the previous year), medications (type and frequency of symptomatic headache medication used in the preceding 31 days, and currently used preventative medications), out of pocket costs (headache-related healthcare expenses within the previous three months, not covered by health insurance), barriers to accessing care (questions on self-recognition of migraine, diagnosis ever of migraine from a healthcare provider, difficulties in accessing a healthcare provider for headache, and any previous cessations of symptomatic, and/or, preventative migraine therapy, with the reasons why, all based on previous studies [15, 16]), and informal care needs (questions from European HIROZON-funded studies by co-author ZA on unpaid care from family or friends, and, if so, how many hours per week [not yet published]).

### Sample size estimation

We estimated the sample size needed to establish migraine prevalence as *N* = 1,750, basing the calculation on the estimated prevalence of migraine for Australia of 0.18 from the Institute of Health Metrics and Evaluation [17], with a relative 10% margin for error. We estimated the number of mail outs needed as *N* = 19,445, assuming that 90% of households would have at least one eligible adult (see below), but a response rate of only 10%. We inflated this to *N* = 20,000 in anticipation that some study letters would inadvertently be sent to non-deliverable addresses.

### Mail out

We outsourced the mail out (including printing invitation letters and questionnaires, addressing envelopes, inserting reply-paid envelopes, and oversight of the process) to Direct Mail Solutions, a well-established company.

### Inclusion criteria

Potentially eligible participants were adults aged 18 years and over. From these, only the person who had most recently had their birthday was asked to respond.

Respondents needed to opt in as participants within the study timeframe, either by entering data directly into the secure online Research Electronic Data Capture (REDCap) platform, accessed via a QR code or weblink included in the study invitation letter, or by returning their hard-copy questionnaires by reply-paid post, with researchers entering the responses into the platform.

**Fig. 1:**
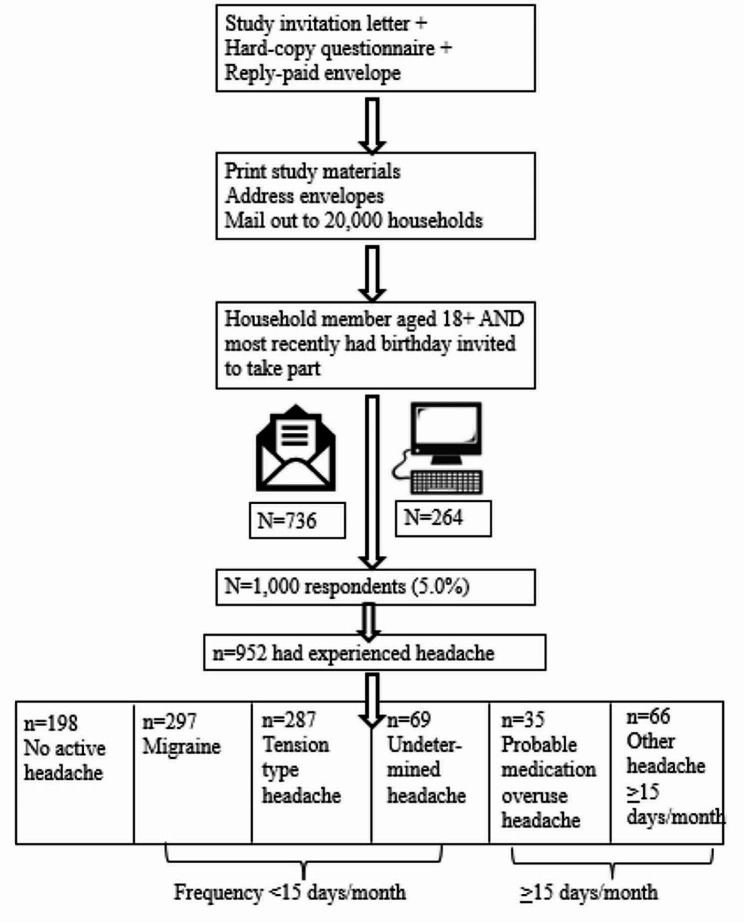
Study workflow

Please see Fig. 1 for the study workflow.

### Data management

Study data were collected and managed using the REDCap electronic data capture tool hosted and managed by Helix (Monash University) [18, 19]. REDCap is a secure, web-based software platform designed to support data capture for research studies, providing: (1) an intuitive interface for data capture; (2) audit trails for tracking data manipulation and export procedures; (3) automated export procedures for seamless data downloads to common statistical packages; and (4) procedures for data integration and interoperability with external sources.

### Diagnoses

Participants reporting any headache within the preceding year were considered to have an active headache disorder, with all others considered to be headache-free. Diagnoses were made during analysis, and not at the time of data collection, using the HARDSHIP algorithm [11], applied to the most bothersome headache type when more than one type was reported. The algorithm first identified those reporting headache on ≥ 15 days/month, diagnosing pMOH when acute medication use on ≥ 10 days/month was also reported and otherwise “other headache on ≥ 15 days/month” (other H15+). In all others with active headache disorder (episodic headache), the algorithm diagnosed, in hierarchical order, definite migraine, definite TTH, probable migraine, probable TTH, and unclassified. Used in this way, the algorithm identifies migraine (definite or probable) with a sensitivity > 70% and a specificity > 70% [12]. 

### Data analysis

Analysis included all participants who answered at least one question.

Participation rate and preferred response method were assessed with reference to age, gender and state. Categorical variables were summarised using frequency and percentage. Continuous variables with approximately normal distribution were summarised using mean and standard deviation (SD), or, otherwise, median and interquartile range (IQR). Prevalence estimates of each headache type were adjusted according to the age and gender distributions of each state. A bootstrapping method with 1,000 iterations was used to estimate variances and calculate 95% confidence intervals (CIs). In the analysis, we combined definite and probable cases of migraine, as well as definite and probable cases of TTH, respectively.

Missing data were summarised using frequency and percentage. The missingness of data was assessed using Little’s chi-squared test for missing completely at random test or covariate-dependent missingness. Since missingness of data was found to be dependent on age, gender, and socioeconomic status (represented in Index of Relative Socio-economic Advantage and Disadvantage [IRSAD] quintile), these covariates were adjusted in the analyses, where applicable.

Statistical significance was set at *p* < 0.05. Holm-Bonferroni’s method was used to control for 5% family-wise error rate in subgroup pairwise comparison, if applicable. All statistical analyses were performed using *Stata* version 16.1 (StataCorp).

### Ethics

The study was granted multisite ethics approval by the Alfred Hospital Ethics Committee (HREC reference: 87,013, Local number: 305/22). Governance approval was granted by the Offices for Research at the individual study sites.

Participation was voluntary, requiring respondents to opt in, with consent therefore presumed.

Only non-identifiable data were captured and therefore participants could not be identified from their responses.

## Results

The study launched on 27 January 2023, mail out was completed over a one-week period, and the study closed to responses on 31 March 2023.

### Respondent characteristics

A total of 1,000 eligible responses were received, the majority (*n* = 736, 73.6%) via reply-paid mail (hard-copy questionnaires), with females and younger age groups significantly more likely to respond online (Table 1). The overall participation rate was therefore 5.0% (1,000/20,000). Participation rates were higher from LGAs with relatively older populations (for every 10 years increase in age, RoM = 1.04, 95% CI: 1.02–1.05, *p* < 0.001). LGAs with middle-level socioeconomic status (IRSAD third quintile) had higher participation rates than those with lower or higher socioeconomic status after adjustment for age (Table 2). Overall, participants’ median age was 60 years (IQR 44–71), and 64.7% were female. English was the most common language spoken at home (93%). Only 1.5% of participants identified as Aboriginal or Torres Strait Islander people (Table 1). These characteristics differed substantially from those of the general Australian population (median age 38 years, 50.7% female, 72% speaking only English at home and 3.2% identifying as Aboriginal or Torres Strait Islander people [20]). 

**Table 1:** Participants’ characteristics by response method

	Reply-paid Return Mail		Online Questionnaire		p-value^	All Respondents	
N (%)	736	(74)	264	(26)		1000	(100)
Age - median (IQR)^1^	64	(53–73)	43	(33–59)	< 0.001	60	(44–71)
Gender - n (%)					0.013		
Male	266	(36)	70	(27)		336	(34)
Female	465	(63)	182	(69)		647	(65)
Other	2	(0.3)	1	(0.4)		3	(0.3)
Not answered	3	(0.4)	11	(4.2)		14	(1.4)
Language spoken most at home - n (%)					0.85		
English	690	(93.8)	237	(89.7)		927	(92.7)
Other	44	(5.9)	16	(6.1)		60	(6.0)
Not answered	2	(0.3)	11	(4.2)		13	(1.3)
Aboriginal and/or Torres Strait Islander - n (%)					0.51		
Yes	10	(1.4)	5	(1.9)		15	(1.5)
No	713	(97)	248	(94)		961	(96)
Not answered	13	(1.8)	11	(4.2)		24	(2.4)

^ Complete case analysis was performed. For analysing gender, only those identifying as males or females were included. For analysing language spoken most at home, all non-English languages were pooled into a single category.

^1^ 23 responders (9 via mail and 14 online) did not provide age information.

**Table 2:** Pairwise comparisons of associations between IRSAD quintiles and participation rate

IRSAD Quintiles	RoM	95% CI	HB corrected p-value
2nd vs. 1^st^	1.21	(1.09–1.34)	0.002
3rd vs. 1^st^	1.42	(1.28–1.57)	< 0.001
4th vs. 1^st^	1.13	(1.02–1.25)	0.099
5th vs. 1^st^	1.18	(1.08–1.30)	0.002
3rd vs. 2^nd^	1.18	(1.09–1.27)	< 0.001
4th vs. 2^nd^	0.93	(0.86–1.01)	0.26
5th vs. 2^nd^	0.98	(0.92–1.05)	0.58
4th vs. 3^rd^	0.79	(0.73–0.86)	< 0.001
5th vs. 3^rd^	0.83	(0.78–0.89)	< 0.001
5th vs. 4^th^	1.05	(0.99–1.12)	0.26

CI, confidence interval; HB, Holm-Bonferroni; IRSAD, Index of Relative Socio-economic Advantage and Disadvantage; RoM, ratio of means.

### Diagnoses

Of the 1,000 eligible responses received, 22 participants were excluded from analysis, six because they reported headache-ever but did not answer whether they had headache in the last 12 months, and 16 because they did not answer the questionnaire. Among 978 participants with valid HARDSHIP questionnaire responses, the vast majority (*n* = 946, 96.7%) reported headache-ever (lifetime prevalence), and most (*n* = 754, 77.1%) reported an active headache disorder (1-year prevalence). The raw 1-year prevalences for each headache type were 3.6% (*n* = 35) for pMOH, 6.8% (*n* = 66) for other H15+, 7.8% (*n* = 76) for definite migraine, 13.6% (*n* = 133) for definite TTH, 22.6% (*n* = 221) for probable migraine, 15.8% (*n* = 154) probable TTH, and 7.1% (*n* = 69) for unclassified headache. Raw prevalences by age, gender, and State are presented in Table 3.

**Table 3:** Raw prevalences by gender and state

n (%)	Probable MoH		Other headache on ≥ 15 days/month		Definite migraine		Probable migraine		Definite TTH		Probable TTH		Unclassified headache		No active headache		Total
New South Wales																	
Female	12	(3.6)	26	(7.7)	23	(6.8)	86	(25.6)	56	(16.7)	54	(16.1)	28	(8.3)	51	(15.2)	336
Male	3	(1.6)	12	(6.3)	11	(5.8)	33	(17.5)	13	(6.9)	35	(18.5)	12	(6.3)	70	(37.0)	189
**Victoria**																	
Female	13	(4.4)	20	(6.7)	29	(9.7)	73	(24.5)	52	(17.4)	39	(13.1)	19	(6.4)	53	(17.8)	298
Male	6	(4.3)	7	(5.0)	13	(9.3)	26	(18.6)	11	(7.9)	23	(16.4)	8	(5.7)	46	(32.9)	140

10 participants reported residence in other states, and 5 participants with ‘other’ (*n* = 3) or missing (*n* = 2) sex information were not shown

MOH, medication-overuse headache; TTH, tension-type headache

Adjusted for sampling weights, age, gender, and State, 1-year prevalence estimates for each headache type were 3.5% (95% CI: 2.2–5.5%) for pMOH, 8.6% (95% CI: 6.6–11.0%) for other H15+, 35.0% (95% CI: 30.9–39.2%) for definite + probable migraine, and 31.0% (27.3–34.9%) for definite + probable TTH, with 6.3% unclassified headache.

Further analyses were not considered appropriate in view of the very low participation rate.

## Discussion

This pilot study revealed that mailed self-reporting headache epidemiological questionnaires yielded low participant engagement, indicating that alternative methods are needed for collecting reliable data from the Australian general population. The bias towards those living with bothersome headache was almost inevitable, given the participating proportion of only 5.0%. The high median age of 60 years and the high female proportion (65%) were clear evidence of bias. The estimated 1-year prevalence of migraine, more than double that expected from estimates in other high-income countries [2, 21], was almost certainly a result of such bias. Therefore, while the data gathered from this study represent the largest body of evidence relating to migraine in the adult Australian general population to date, the low response rate introduces a high risk of non-response bias and selection bias, which means all other findings are of highly questionable value.

In anticipation of the possibility of a low participation rate, we took a number of pre-emptive measures: we offered both hard-copy and online versions of the questionnaire; engaged a consumer advocate to inform the construction of an appealing study invitation letter that was in colour, included logos of participating institutions, photographs of the researchers, and clear explanation of why the study was important; created a study webpage hosted on an institutional website to provide background information about the study and the researchers involved; created a Twitter/X account (@aheadstudy2022) to keep members of the public informed on study progress; and created a study video that was displayed on the study webpage and on the landing page of the online questionnaire to explain the study in an engaging visual format. The study launch attracted substantial media attention, with the lead investigator (EF) invited to promote it on nine radio stations across the country, achieving two primetime news bulletins, and it appeared in print in Australia’s most widely read newspaper. All, it seems, were insufficient.

Our participation rate was lower than those achieved by other international population-based epidemiology studies using mailed questionnaires in high-income countries [22]. The EUROLIGHT project was a survey-based headache epidemiology study conducted across 10 European countries using different sampling methods [22]. As in our study, Germany and Italy distributed study questionnaires to the general population via regular post, requested return via reply-paid envelopes, and sent no reminders. Participation rates were 11.3% and 14.3%, respectively. Luxembourg also had similar methodology with the exception that a reminder was sent to non-responders one month later; their responder rate was 31.1%. One explanation for the higher participation rates from these countries was that their study questionnaires were addressed to specific individuals, who had been selected from lists provided by local authorities to ensure representative sampling of the general population. Our study addressed letters impersonally ‘To the householder’. We were unable to access a list of individuals’ names and addresses that were representative of the general population as the responsible government department was overseeing the federal election at the time of our study. In addition to addressing questionnaires to specific individuals and introducing follow-up approaches (as done in the EUROLIGHT project), future research may need to consider other tactics, such as pre-notifications, so that arrival of the questionnaire is expected, with its purpose already understood, incentives to respond (monetary or non-monetary, as used in the Australian Longitudinal Study on Women’s Health) [23], remainders chasing non-responders, and availability of other language versions of the questionnaire. Each of these will increase the resources needed, and none is guaranteed to increase the participating proportion, or improve reliability (each may introduce other biases). A shorter questionnaire may yield a higher response rate [24], but limit the value of the study.

Very clearly, a different design will be needed for a nationwide epidemiological study. For many diseases, nationwide prevalence data can be drawn from administrative datasets. For example, acute stroke in almost all cases results in hospital contact, and multiple sclerosis has disease-specific therapies. Prevalence of these disorders may reasonably be inferred from hospital discharge codes and prescription datasets, respectively. Migraine attacks rarely present to hospital, and only 22% of specialists, such as neurologists, have used the national healthcare record that aims to store key health information [25]. In Australia, most oral migraine preventative drugs must be prescribed privately, since the universal pharmaceutical insurance scheme (Australian Pharmaceutical Benefits Scheme) does not list migraine as an indication for relevant antiseizure medications, mood stabilisers, antihypertensives, and so on, and private script data are not available for data linkage studies [26]. Migraine-specific therapies, such as triptans and anti-CGRP monoclonal antibodies, are included in Australia’s Pharmaceutical Benefits Scheme, but their use is restricted to people who meet specific inclusion and exclusion criteria. For all these reasons, a data linkage approach would grossly underestimate migraine prevalence, and would not account for undiagnosed cases of migraine in the community. Other countries have administered structured or semi-structured headache epidemiological questionnaires via telephone with trained lay reviewers [27]. However, in Australia the publicly available record of telephone numbers does not include residential addresses that have ‘opted out’ of listing, potentially resulting in a non-representative sample, and it might be anticipated that the vast majority of study calls, coming from an unknown number, would be rejected by prospective participants [28]. Another approach would be to compile a study sample that was demographically and geographically representative of the general population via market research companies [29]. Such a sample might receive monetary or other incentives to complete questionnaires, improving response rates but also introducing bias among those choosing to participate.

Following on from our study, it would seem the most pragmatic approach for collecting Australian headache prevalence data would be to include validated diagnostic questions in an upcoming cycle of the National Health Survey (NHS) [7]. The NHS is conducted every few years by the Australian Bureau of Statistics. Over a few weeks’ period, trained interviewers conduct face-to-face semi-structured interviews with > 20,000 households that are demographically representative of the general population. This type of study is well outside the budget of most research groups. In recognition of this, the NHS selects a limited number of conditions for specific examination with each cycle. Given the difficulties in establishing prevalence of headache types (e.g., migraine, pMOH) through self-report questionnaires, as comprehensively demonstrated in our study, and by other methods, coupled with the substantial burden of headache evidenced elsewhere, there is an extremely compelling case to include at least migraine in an upcoming NHS.

### Limitations

First and foremost, as already discussed, this study was limited by the low participation rate, with inevitable biases. The findings with regard to prevalence are neither reliable nor generalizable. This study was, nonetheless, informative as a pilot study to test the methodology before investment of resources in a definitive study.

## Conclusion

The mailed self-report questionnaire had a very low participation rate, clearly indicating that it would not be a suitable methodology for a future nationwide epidemiological study. Instead, these findings strongly support the inclusion of dedicated migraine diagnostic questions in an epidemiological study with higher participant engagement, for example, the National Health Survey. The arguments for doing this are very compelling.

## Appendix 1: the AHEAD Study Pilot Questionnaire (Hard Copy version)

Please note: the QR code and weblink to the online version of the questionnaire are now disabled. Please contact lead investigator emma.foster@monash.edu should you wish to view the online version of the questionnaire.

### Electronic supplementary material

Below is the link to the electronic supplementary material.

Supplementary Material 1

## Data Availability

No datasets were generated or analysed during the current study.
