# Supplementary material for: Australian Headache Epidemiology Data (AHEAD): a pilot study to assess sampling and engagement methodology for a nationwide population-based survey
Source: J Headache Pain. 2024 May 6;25(1):71. doi: 10.1186/s10194-024-01773-8 (PMC11075354; doi:10.1186/s10194-024-01773-8)
Supplement: Supplementary file 1 — Supplementary Material 1 [file 10194_2024_1773_MOESM1_ESM.pdf]

AHEAD

Headache in Australia

Australian Headache Epidemiology Data Study

This research is funded by a grant from the Brain Foundation, Lundbeck, and Prince of Wales Hospital Foundation

Invitation to participate in our study

Scan this QR code to access the survey

Contact us (through either of the following) -

Email: christine.cormack@health.nsw.gov.au

Telephone: +61 2 9382 3912

Post: Attention: Chris Cormack, Institute of Neurological Sciences, Prince of Wales Hospital,
Level 2, High Street Building, High Street, Randwick NSW 2031

For more information on this study, please visit:
https://www.monash.edu/ahead-study

Hi there!

We are researchers from Monash University, Alfred Health, UNSW, and Prince of Wales Hospital, undertaking the largest survey about headache in Australia. We are trying to find out how many people have headache and how it affects them.

This important knowledge will shape Australian headache care in the coming years. We invite the member of your household who is **aged 18 or over AND most recently had their birthday** to participate.

Participation is completely voluntary. You do not have to take part if you do not want to.

Responses are anonymous. That means there is no way for anyone (including the researchers) to personally identify people who choose to take part in the study.

Even if the selected member of your household (i.e., who is 18 or over and most recently had their birthday) does not get headaches, we would appreciate their responses. This is in order to provide a balanced view of who does and who does not get headaches.
If you'd like to take part, please do so with ONE of the following options:

1) Complete this booklet and return it in the reply-paid envelope, OR
2) Scan the QR code above and follow the prompts, OR
3) Using a web browser, enter: https://redcap.link/AHEADstudy and follow the prompts.

The survey will take between 5-15 minutes to complete.

If you'd like to participate, please do so before the closing date: **31 March 2023**

Thank you for helping us with this important research.

MONASH University AlfredHealth UNSW SYDNEY Prince of Wales Hospital & Community Health Services

DEMOGRAPHICS

We use this information to make sure we have a representative sample of the Australian population. We also use it to see if different groups are impacted by headaches differently.

1. What is the postcode of your home address? __________________

2. What is your age in years? __________________

3. What is your gender? (Please choose one option)
   - ☐ Male
   - ☐ Female
   - ☐ Other

4. Do you identify as an Aboriginal and/or Torres Strait Islander person? (Please choose one option)
   - ☐ Neither
   - ☐ Yes, Aboriginal person
   - ☐ Yes, Torres Strait Islander person
   - ☐ Yes, both Aboriginal and Torres Strait Islander person

5. What language do you speak **most** at home? (Please choose one option)
   - ☐ English
   - ☐ Arabic
   - ☐ Cantonese
   - ☐ Greek
   - ☐ Hindi
   - ☐ Italian
   - ☐ Mandarin
   - ☐ Punjabi
   - ☐ Spanish
   - ☐ Tagalog
   - ☐ Vietnamese
   - ☐ Other ______________________________

SCREENING QUESTIONS

6. Have you ever had a headache in your **lifetime**?
   - ☐ No. Please go straight to the EQ5D Health Questionnaire on page 9.
   - ☐ Yes

7. Have you had a headache in **the last 12 months**?
   - ☐ No. Please go straight to Question 29a on page 7.
   - ☐ Yes

8. During **the last 30 days**, on how many of these days did you have a headache? (please write the number of days between 0 and 30)
   - __________ days

HEADACHE QUESTIONS

With regards to your headache (if you have more than one type of headache, with regards to **the most bothersome** type):

9. **How long** does this headache usually last? *Please answer how long it lasts if you do NOT take medication for it. If the headache goes away during sleep, count the time until you wake up without it.*
   - ____ minutes,
   - ____ hours, or
   - ____ days, or
   - ☐ Never goes away

10. There are many ways of describing a headache, but most are either throbbing or pressing.

    Which **best** describes the pain?
   - ☐ Throbbing or pulsating (this means varying in time with the heartbeat)
   - ☐ Pressing, squeezing, or tightening

11. Is the pain usually on only **one side** of the head?
   - ☐ No ☐ Yes

12. How **bad** is the pain?
   - ☐ Mild ☐ Moderate ☐ Severe

13. Does **exercise** (like walking or climbing stairs) tend to make it worse?
   - ☐ No ☐ Yes

14. How does this headache affect your **ability** to do day-to-day activities?
   - ☐ Can do everything as normal
   - ☐ Cannot do some things
   - ☐ Can do nothing

HEADACHE QUESTIONS (continued)

With regards to your headache (if you have more than one type of headache, with regards to **the most bothersome** type):

15. With this headache, do you usually feel nausea (as though you may vomit or throw up?) ☐ No ☐ Yes

16. With this headache, do you usually vomit (throw up)? ☐ No ☐ Yes

17. With this headache, does daylight or other lighting bother you? In other words, do you prefer to be in the dark?
This question refers to ordinary levels of light, not bright lighting.
☐ No
☐ Not sure
☐ Yes

18. With this headache, does noise bother you? In other words, do you prefer to be in the quiet?
This question refers to ordinary levels of noise, not very loud noise.
☐ No
☐ Not sure
☐ Yes

HEADACHE BURDEN

Because of your headaches, in **the last 30 days**, how many days:

19. Could you **not go** to work or school? ________ days

20. Could you do **less than half** your usual amount in your job or schoolwork? (Do not include days you counted in Question 19 where you missed whole days of work or school). ________ days

21. Could you **not do any** household work? (This includes repairs, maintenance, shopping, caring roles) ________ days

22. Could you do **less than half** your usual amount of household work? (Do not include days you counted in any of the previous questions). ________ days

23. Did you **miss** family, social, or leisure activities? ________ days

HEALTHCARE SERVICE

24. Have you received advice about your headaches from a healthcare provider in **the last year**?

☐ No. Please go to Question 25.

☐ Yes. Please tick all the healthcare providers you have seen (in person, via telehealth, or via telephone) and write next to each how many times you consulted them about **headaches** in **the last year:**

☐ Chiropractor ____________

☐ Ear, nose, and throat (ENT) doctor ____________

☐ Eye specialist (Ophthalmologist, Optometrist) ____________

☐ GP (Local or Family doctor) ____________

☐ Neurologist ____________

☐ Nurse ____________

☐ Osteopath ____________

☐ Pain specialist ____________

☐ Physiotherapist ____________

☐ Other ______________________________

TESTS

25. To investigate your **headaches**, have you had any tests in **the last year**?

☐ No. Please go to Question 26.

☐ Yes. Please tick all the tests you have had:

☐ MRI brain scan
☐ CT brain scan
☐ X-rays or other scans of the neck or cervical spine
☐ Eye tests (for glasses)
☐ Blood tests

☐ Other: ______________________________

____

HOSPITAL ATTENDANCES

In **the last year, because of your headaches**, have you:

26. Visited a hospital emergency department (accident and emergency)?

☐ No

☐ Yes. Number of visits in **the last year**: ______

27. Been admitted to hospital **because of your headaches**?

☐ No

☐ Yes. Number of **days** spent in hospital for headache in the last year: ______

MEDICATIONS

28. Did you take **any** medications (prescribed or over the counter) to relieve your headache pain or other headache symptoms in **the last month (31 days)?**

☐ No. Please go to Question 29a.

☐ Yes. Please tick **all** the medications and indicate how many days you used them in **the last month**:

**Name of medication**	**Number of days (0-31) used in last month**
☐ Eletriptan (Relpax)	
☐ Naratriptan (Naramig)	
☐ Rizatriptan (Maxalt)	
☐ Sumatriptan (Imigran)	
☐ Zomitriptan (Zomig)	
☐ Ergotamine (Cafergot, Ergodryl)	
☐ Domperidone (Motilium)	
☐ Metoclopramide (Maxolon, Primperan)	
☐ Ondansetron (Zofran)	
☐ Prochlorperazine (Stemetil, Stemzine)	
☐ Aspirin	
☐ Diclofenac (Voltaren)	
☐ Ibuprofen (Nurofen, Advil, Rafen, etc.)	
☐ Indomethacin (Indocid, Arthrexin)	
☐ Ketoprofen (Orudis, Ketocid)	
☐ Ketorolac (Toradol)	
☐ Mefenamic acid (Ponstan)	
☐ Naproxen (Naprosyn)	
☐ Paracetamol (Panadol, Panamax)	
☐ Paracetamol **with** aspirin **and** caffeine (Excedrin)	
☐ Paracetamol **with** caffeine (Panadol extra)	
☐ Aspirin **with** codeine (Aspalgin, Codral Forte, Disprin Forte)	
☐ Ibuprofen **with** Paracetamol (Paracetafen, Nuromol, Maxigesic)	
☐ Codeine	
☐ Endone	
☐ Ibuprofen **with** codeine (Brufen plus, Panafen plus, Advil plus, Nurofen plus)	
☐ Paracetamol **with** codeine (Codalgin, Panadeine, Panadeine forte, Mersyndol)	
☐ Tapentadol (Palexia)	
☐ Tramadol (Tramal)	
☐ Other. Please list other treatments you use to treat headache symptoms here. (We will ask about preventative (daily) medications in the next question).<br>____<br>____	

MEDICATIONS (continued)

29a. Do you **currently** take any preventative headache medication? (These are usually taken daily.)

☐ No. Please go to Question 29b.

☐ Yes. Please select them below, as well as how long you have been taking them (in **months**).

Name of medication	Duration of use (number of months)
☐ Amitriptyline (Endep)	
☐ Candesartan (Atacand)	
☐ Pizotifen (Sandomigran)	
☐ Propranolol (Inderal)	
☐ Topiramate (Topamax)	

☐ Other ______________________

____

29b. Do you **currently** take injections or use a device therapy for headache?

☐ No. Please go to Question 30.

☐ Yes. Please select them below, as well as how long you have been taking them (in **months**).

Name of therapy	Duration of use (number of months)
☐ Botox® injections	
☐ CGRP injections	
☐ Aimovig®	
☐ Emgality®	
☐ Ajovy®	
☐ Device therapy (e.g., Cefaly®, gammaCore™)	

☐ Other ______________________

____

COST

30. In **the last 3 months**, approximately how much have all these treatments and appointments in Questions 24-29b cost you (after Medicare and private health reimbursement)? ______________________

YOUR PERSPECTIVE

31. Do you have migraine? ☐ No ☐ Yes

32. Have you ever been told by a healthcare provider that you have migraine? ☐ No ☐ Yes

33. Have you had difficulty consulting a healthcare provider regarding your headache? ☐ No ☐ Yes

If yes, please tick all that apply:

- ☐ Difficulty finding a headache-focused healthcare provider
- ☐ Long wait times to see a headache-focused healthcare provider
- ☐ Cost to see a headache-focused healthcare provider
- ☐ Distance to visit a headache-focused healthcare provider
- ☐ Language barrier
- ☐ Other ____________________

34a. Have you had to stop taking an **acute** (as-needed headache pain relief) medication because:

i. It did not work?	☐ No	☐ Yes
ii. It caused side effects?	☐ No	☐ Yes**
iii. It cost too much?	☐ No	☐ Yes
iv. Other ____________________		

34b. Have you had to stop taking a **preventative** (daily) headache medication because:

i. It did not work?	☐ No	☐ Yes
ii. It caused side effects?	☐ No	☐ Yes**
iii. It cost too much?	☐ No	☐ Yes
iv. Other ____________________		

***Optional: If you experienced side effects to acute and/or preventative medications, and you would like to provide further details, please do so below:*

____

____

INFORMAL CARE NEEDS

35. **Because of your headaches**, do you receive informal, unpaid care from family members, relatives, or friends?

- ☐ No. Please go to the EQ5D Health Questionnaire on Page 9.
- ☐ Yes

36. How many **hours** of informal, unpaid care **per week** do you receive from family members, relatives, or friends? ____________

EQ-5D-5L Cover Page

English (Australia) version

(Blank for copyright reasons)

EQ-5D-5L Questions

English (Australia) version

(Blank for copyright reasons)

EQ-5D-5L Visual Acuity Scale

English (Australia) version

(Blank for copyright reasons)

AHEAD Headache in Australia

Australian Headache Epidemiology Data Study

This research is funded by a grant from the Brain Foundation, Lundbeck, and Prince of Wales Hospital Foundation

Study Summary

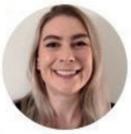
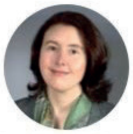
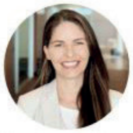
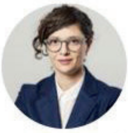
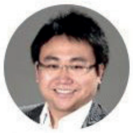
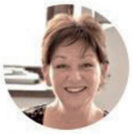
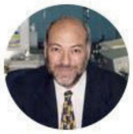

Dr Emma Foster	Dr Elspeth Hutton	Prof Claire Wakefield	A/Prof Zanfina Ademi	Dr Ben Chen	Chris Cormack	A/Prof Alessandro Zagami
Neurologist & Researcher (VIC)	Neurologist & Researcher (VIC)	Patient Advocate (NSW)	Senior Health Economist (VIC)	Senior Biostatistician (VIC)	Research Coordinator (NSW)	Neurologist & Researcher (NSW)

We are a group of Australian Headache Specialists and Researchers. We are conducting an important survey that will help close the largest knowledge gap in Australian Headache Medicine.

Worldwide, migraine is the second leading cause of years lived with disability.

Questions we aim to answer include:

- How common is migraine in Australia?
- How does it affect our lives, work, and families?
- Can we make this better?

What? The survey includes 42 questions and it takes about 5-15 minutes to complete. Participation is **completely voluntary**. If you do not wish to take part, you do not have to. The survey is **anonymous.** You will NOT be asked to provide your name, date of birth, address, or any other information that can identify you.

Why? The survey data will guide future headache and migraine research and healthcare planning. We hope that this will bring meaningful change to individuals, their families, and our communities.

How? You can access the survey via the QR code in the top right-hand corner of this letter, via this website link: https://redcap.link/AHEADstudy or by completing the enclosed paper-based survey and returning it via reply-paid post.

For more information, please visit our webpage: https://www.monash.edu/ahead-study or contact our Research Coordinator Ms Chris Cormack, Ph: 02 9382 3912 Email: christine.cormack@health.nsw.gov.au

Thank you for helping us with this important research.

Monash University · AlfredHealth · UNSW Sydney · Prince of Wales Hospital & Community Health Services
